# Examination of Quaternary Ammonium Compound Resistance in *Proteus mirabilis* Isolated from Cooked Meat Products in China

**DOI:** 10.3389/fmicb.2017.02417

**Published:** 2017-12-11

**Authors:** Xiaobing Jiang, Tao Yu, Lei Liu, Yi Li, Kun Zhang, Hailei Wang, Lei Shi

**Affiliations:** ^1^College of Life Sciences, Henan Normal University, Xinxiang, China; ^2^College of Life Science and Technology, Xinxiang University, Xinxiang, China; ^3^Institute of Food Safety and Nutrition, Jinan University, Guangzhou, China

**Keywords:** *Proteus mirabilis*, *qac* genes, integron, benzalkonium chloride, resistance

## Abstract

The aim of this study was to examine the presence of genes responsible for resistance to quaternary ammonium compounds (QACs) and the association of *qac* genes with class 1 integrons in *Proteus mirabilis* isolated from cooked meat products. A total of 52 *P. mirabilis* isolates (29.2%) were detected from 178 samples, and their minimum inhibitory concentrations (MICs) of benzalkonium chloride (BC) ranged from 4 to >32 μg/mL. The isolates with BC MICs of 24 μg/mL were observed most frequently. PCR assays indicated that *mdfA, ydgE*/*ydgF, qacE, qacE*Δ1, *emrE, sugE(c)*, and *sugE(p)* were commonly present (32.7%–100%) in these isolates, but *qacH* was less prevalent (3.8%). Five groups of resistance gene cassettes were identified in 10 *intI1*-positive isolates. An unusual gene cassette array *dfrA32*-*ereA*-*aadA2* was found in one foodborne isolate of *P. mirabilis*. Two isolates harbored *qacH*- and *sul3*- associated non-classic integrons: *aadA2-cmlA1-aadA1-qacH-*IS*440-sul3* and a new arrangement *dfrA32*-*ereA1*-*aadA2-cmlA1-aadA1-qacH-*IS*440-sul3*, which is first reported in *P. mirabilis*. Non-classic class 1 integrons were located on conjugative plasmids of 100 kb in two tested isolates. Our data showed that the QAC resistance genes were commonly present among *P. mirabilis* isolates from cooked meats and *qacH* was associated with non-classic class 1 integrons. The creation of transconjugants demonstrated that *qacH*-associated non-classic class 1 integrons were located on conjugative plasmids and therefore could facilitate the co-dissemination of disinfectant and antimicrobial resistance genes among bacteria, an increasing area of concern.

## Introduction

*Proteus mirabilis*, widely distributed in the natural environment, is a member of Enterobacteriaceae family. As an opportunistic pathogen, *P. mirabilis* can cause urinary tract and wound infections. In addition, it can also contaminate meat (Kim et al., 2005; Wong et al., 2013), vegetables (Uzeh et al., 2009), and seafood (González-Rodríguez et al., 2002), and has been associated with food poisoning (Wang et al., 2010). Previous studies focused on the antimicrobial resistance and the distribution of resistance genes among foodborne *P. mirabilis* (Kim et al., 2005; Wong et al., 2013). Unlike other foodborne bacteria (Zou et al., 2014; Zhang et al., 2016), little data have been reported on the disinfectant resistance and molecular mechanisms underlying resistance in foodborne *P. mirabilis*. Disinfectants have the ability to co-select for antimicrobial resistance when these traits are genetically linked (Chapman, 2003). Moreover, much evidence has shown that disinfectant and antimicrobial resistance genes can be co-transferred between bacteria via horizontal gene transfer (Zhao et al., 2009; Call et al., 2010; Sáenz et al., 2010; Partridge et al., 2012), which poses a risk to public health.

Benzalkonium chloride (BC), an important representative of quaternary ammonium compounds (QACs), is used extensively as a disinfectant in the food processing environment to prevent the growth of microorganisms and to ensure the microbiological safety of food products. Its widespread use, however, may impose a selective pressure for resistant strains of bacteria (Cruz and Fletcher, 2012). In the past decades, resistance to BC (resistant breakpoints for BC were >30 μg/mL in Sidhu et al. (2002) and ≥20 μg/mL in Meier et al. (2017)) has been reported in many bacterial isolates from food and food processing plants (Sidhu et al., 2002; Meier et al., 2017).

Efflux pumps, QacE, QacEΔ1, QacF, QacG, QacH/I, and SugE(p), which contribute for BC resistance, have been identified in Gram-negative bacteria (Zou et al., 2014). They are members of the small multidrug resistance (SMR) family and are generally located on mobile genetic elements, such as integrons and plasmids (Zou et al., 2014). *qac* are closely associated with class 1 integrons. *qacE* is located in the 3′-conserved segment (CS) of class 1 integrons and *qacE*Δ1, a deletion derivative of *qacE*, confers increased resistance to BC (Kazama et al., 1998). *qacF* shows a high degree of similarity (67.8% identity) with *qacE*. Class 1 integrons carrying *qacG* have been found in Gram-negative bacteria (Chu et al., 2001; Partridge et al., 2012). *qacH*, which was first identified in *Staphylococcus* (Heir et al., 1998), has been observed frequently in Gram-negative bacteria (Hegstad et al., 2010) and it confers a broader resistance phenotype compared with *qacG* (Heir et al., 1998). *qacH* is usually recognized as an important component of class 1 integron that lacks the normal 3′-CS region (i.e., a “non-classic class 1 integron”), which has been detected in many species of Enterobacteriaceae (Antunes et al., 2007; Chang et al., 2009, 2011; Sáenz et al., 2010; Farkas et al., 2016). Several studies found *qacH* gene and *β*-lactamase genes (*bla*_IMP-15_, *bla*_GES-1_, *bla*_GES-5_, and *bla*_OXA-2_) linked to class 1 integrons from *Pseudomonas aeruginosa* clinical isolates (Garza-Ramos et al., 2008, 2010; Viedma et al., 2009). *qacH*-*aadB* has been reported from environmental bacteria, including *Paracoccus versutus, Brevundimonas diminuta, Brachymonas denitrificans, Stenotrophomonas acidaminiphila*, and *Psychrobacter* spp. (Li et al., 2009) and *qacH*-*aadA8* from *Vibrio cholerae* (Ceccarelli et al., 2006). Additionally, *qacH* located on a novel transposon Tn*6188* was found in *Listeria monocytogenes* (Müller et al., 2013). In several studies, *qacH* in Enterobacteriaceae was renamed as *qacI* to distinguish it from *qacH* from *Staphylococcus* (Naas et al., 2001; Curiao et al., 2011). For this paper, we still use the gene name “*qacH*”. *qacH* exhibits 91.6% similarity with the sequence of *qacF*. Finally, *sugE(p)* is frequently present on multidrug resistance plasmids, that have been reported in *Escherichia coli* and *Salmonella* (Zhao et al., 2009; Call et al., 2010).

Five chromosome-encoded efflux pump genes (*sugE(c), emrE, mdfA*, and *ydgE/ydgF*) have been reported to confer resistance to BC (Bay and Turner, 2009; Zou et al., 2014). In addition to *mdfA* encoding a multidrug resistance efflux pump belonging to the major facilitator superfamily (MFS), the remaining genes encode the SMR family efflux pumps.

The aims of this study were to assess the BC resistance and investigate the presence of disinfectant resistance genes and the association of *qac* genes with class 1 integrons among the *P. mirabilis* isolates from cooked meat products in China.

## Materials and Methods

### *P. mirabilis* Isolates

Between January and September 2015, 178 samples (250 g) of cooked meat products, including roasted meats (*n* = 67) and sauced meats (*n* = 111), were purchased from supermarkets and cooked meat shops in Xinxiang, a city of Henan. Six local supermarkets and ten cooked meat shops were randomly selected and each site was visited once. Thirteen samples were collected from each supermarket and ten samples from each cooked meat shops. Samples were transported to the laboratory in an icebox and were processed immediately for bacterial isolation. According to the previous study, the traditional *Salmonella* isolation protocol was modified to isolate *P. mirabilis* (Wong et al., 2013). Briefly, a rinse was performed by adding 25 g of sample to 225 mL of buffer peptone water (BPW; Huankai Ltd., Guangzhou, Guangdong, China) in sterile lateral filter bags with thorough mixing by using a homogenizer (BagMixer lab blender 400; Interscience, Saint-Nom-La-Breteche, France). These samples were then incubated at 37°C for 18 h. Pre-enriched sample (1 mL) was inoculated into 10 mL of tetrathionate broth base (TTB; Huankai) and incubated at 42°C for 24 h. A loop of inoculum was streaked onto xylose lysine deoxycholate agar (XLD; Huankai) and incubated for 24 h at 37°C. Three to four *Salmonella*-like colonies (pink with or without black center on XLD; and yellow with or without black center colonies on XLD were also considered as suspected colonies) were picked and re-streaked on nutrient agar (NA). The isolates with swarming phenotype were identified by using the API 20E bacterial identification system (BioMerieux, Marcy l’Etoile, France). All isolates designated as *P. mirabilis* were additionally analyzed by PCR-based 16S rDNA sequencing using a pair of universal primers 27F/1492R (**Table 1**). Only one isolate from each sample was selected for further characterization.

**Table 1:** Primers used in this study.

Target gene(s) or region	Primer	Sequence (5′–3′)	Annealing temperature (°C)	Amplicon size (bp)	Reference
16S rDNA	27F	AGAGTTTGATCCTGGCTCAG	55	1466	Moreno et al., 2002
	1492R	GGTTACCTTGTTACGACTT			
qacE	qacE-F	AGCCCCATACCTACAAAG	55	194	Gillings, 2010
	qacE-R	AGCTTGCCCCTTCCGC			
*qacE*Δ1	qacEΔ1-F	AAGTAATCGCAACATCCG	49	140	Jiang et al., 2017
	qacEΔ1-R	ATAAGCAACACCGACAGG			
*qacF*	qacF-F	TTCCTTCCGTTGTAGTTGT	52	228	Jiang et al., 2017
	qacF-R	CCTTGGATAGCAGGTTTAG			
*qacG*	qacG-F	GTCGCTGACACTCAAATCG	52	133	Jiang et al., 2017
	qacG-R	GACACCAACAAATCCCCAC			
*qacH*	qacH-F	TTTGGTGAGGTCGTCGCA	54	162	Jiang et al., 2017
	qacH-R	GCCAGCCCAAACAGCATA			
*sugE(p)*	sugE(p)-F	CAATCGCCCAGACAACTT	51	103	Jiang et al., 2017
	sugE(p)-R	GCAAACGCTTCTTTCACC			
*sugE(c)*	sugE(c)-F	CTGCTGGAAGTGGTATGGG	55	226	Zou et al., 2014
	sugE(c)-R	GCATCGGGTTAGCGGACT			
*emrE*	emrE-F	CCTGTTATGGGCGGTAGAC	54	310	Jiang et al., 2017
	emrE-R	TTCGTGCTCACCTTTCCTT			
*mdfA*	mdfA-F	GTCAGGCGTTACTTTTCC	54	596	Jiang et al., 2017
	mdfA-R	GTCACGACCGAGTTCTTT			
*ydgE*	ydgE-F	GGCAATCGTGCTGGAAAT	54	184	Jiang et al., 2017
	ydgE-R	GGCGGCAATACCAAACCC			
*ydgF*	ydgF-F	ATTACCTTGTTTAGCGTTTT	49	139	Jiang et al., 2017
	ydgF-R	GGTTCACCTCCAGTTCAG			
*intI1*	intI1-F	ACGAGCGCAAGGTTTCGGT	50	565	Li et al., 2006
	intI1-R	GAAAGGTCTGGTCATACATG			
*qacEΔ1*-*sul1* region	qac-F	TAGCGAGGGCTTTACTAAGC	55	632	Jiang et al., 2017
	sul1-R	CAGTCCGCCTCAGCAATATC			
variable region	InF	GGCATACAAGCAGCAAGC	52		Sandvang et al., 1998
	InB	AAGCAGACTTGACCTGAT			
*aadA2*	aadA2-F	CATCCCGTGGCGTTATCC	56	370	Jiang et al., 2017
	aadA2-R	CTGGGCAGGTAGGCGTTT			
*cmlA1*	cmlA1-F	CGCCACGGTGTTGTTGTTAT	57	694	Jiang et al., 2017
	cmlA1-R	TTGCCTGCCCATCATTAGTC			
*aadA1*	aadA1-F	CGTAAGGCTTGATGAAACA	52	141	Jiang et al., 2017
	aadA1-R	GGATAACGCCACGGAATG			
IS*440*	IS440-F	TATTCCGATTGAACACCTT	53	299	Jiang et al., 2017
	IS440-R	TATTGGCGTTGATTACAGC			
*sul3*	sul3-F	CGAGATTTCACATCGGTTCC	50	208	Jiang et al., 2017
	sul3-R	TTGCTGCTTTAGTTGAGGCT			

### Determination of Minimum Inhibitory Concentrations (MICs) for BC

The MICs of BC for *P. mirabilis* were determined using the agar dilution method recommended by the Clinical and Laboratory Standards Institute (Clinical and Laboratory Standards Institute, 2012). BC was tested in concentration range of 4–32 μg/mL. *P. mirabilis* suspensions were adjusted to a turbidity equivalent to that of a 0.5 McFarland standard with sterilized saline solution (0.9%) and delivered to the Mueller–Hinton (MH; Huankai) agar containing different concentrations of BC (Aladdin Biochemical Technology Co., Ltd., Shanghai, China). The plates were incubated at 37°C for 24 h. The lowest concentration of BC that prevented growth was considered as the MIC. Each of the tests was done in triplicate. In cases in which not all three replicates had the same results, the MICs were determined once more. *Escherichia coli* ATCC 10536 (a gift from Lijun Zhou, Navy General Hospital, Beijing, China) was used as a quality control strain (the MIC of this strain for BC was 16 μg/mL).

### Detection of QAC Resistance Genes

All isolates were screened by PCR for the presence of *qac* genes, including *qacE, qacE*Δ1, *qacF, qacG, qacH, sugE(p), sugE(c), emrE, mdfA*, and *ydgE*/*ydgF* (**Table 1**). Colonies were transferred to an Eppendorf tube filled with water and boiled to prepared DNA template (Wang et al., 2008). The PCR mixture consisted of 2.5 μL of boiled lysate DNA, 0.6 μM (each) primer, 200 μM deoxynucleoside triphosphate (Takara Bio Inc., Otsu, Shiga, Japan), 1× PCR buffer (Takara), 0.5 U *Taq* DNA polymerase (Takara) in a total volume of 25 μL. The PCR conditions were as follows: initial denaturation at 94°C for 5 min followed by 30 cycles of denaturation at 94°C for 30 s, annealing at different temperatures (between 49 and 55°C depending on the primer set) for 30 s, extension at 72°C for 30 s, and a final extension at 72°C for 7 min. The purified PCR products were sequenced and DNA sequence data were analyzed using the BLAST program^1^.

### Characterization of Class 1 Integrons

All isolates were screened for *intI1* (**Table 1**). Because the occurrence of sulfonamide resistance gene (*sul1*) and quaternary ammonium compounds resistance gene (*qacE*Δ1) is often associated with classic class 1 integrons, presence of *qacE*Δ1-*sul1* region in all *intI1*-positive isolates was tested by using the primers qac-F and sul1-R (**Table 1**). The presence of gene cassettes in variable regions were characterized by PCR using specific primers (**Table 1**). Primers InF and InB were used for classic class 1 gene cassettes amplifications; and primers InF and aadA2-R were used for non-classic class 1 integron amplifications. To determine the genetic structure of non-classic class 1 integrons, a wide variety primers were designed. PCR “primer-walking” strategy was used to amplify overlapped individual fragments using Takara LA *Taq* DNA polymerase to get the complete arrangement. All the obtained amplicons were sequenced on both strands.

### Genetic Locations of *qacH*-Associated Class 1 Integrons

Plasmid DNA was isolated from two *qacH*-positive isolates using TIANpure Mini Plasmid Kit (TIANGEN Biotech Co., Ltd., Beijing, China). To determine the number and size of plasmids, genomic DNA from *qacH*-positive isolates was performed by S1 nuclease (Takara) digestion prior to PFGE. S1-PFGE fragments were transferred onto membranes (Amersham Pharmacia Biotech, Buckinghamshire, United Kingdom) by Southern blot and hybridized with specific probes of *intI1* and *qacH* genes. The probes were made with the DIG-High Prime DNA Labeling and Detection Starter Kit I (Roche Applied Science, Mannheim, Germany).

### Conjugation Experiments

The transfer of plasmids carrying *qacH*-associated class 1 integrons was studied by performing conjugation experiments as described previously (Koo and Woo, 2011). *E. coli* J53Az^r^ (a sodium azide resistant strain) was used as the recipient and isolates containing non-classic class 1 integron severed as donors. Briefly, donor and recipient cells were mixed with each other at 1:10 ratio and incubated at 37°C overnight. Transconjugants were selected on trypticase soy agar (TSA; Huankai) plates containing sodium azide (150 μg/mL; Sinopharm Chemical Reagent Co., Ltd., Shanghai, China), streptomycin (50 μg/mL; Sigma–Aldrich, St. Louis, MO, United States) and chloramphenicol (16 μg/mL; Sigma–Aldrich). PCR was used to confirm that the transconjugants carried the same resistance genes as their donors.

### Statistical Analysis

The statistical package SPSS 15.0 (SPSS Inc., Chicago, IL, United States) was used, and the two-tailed paired Student’s *t*-test was applied to determine the significance of differences. A *P*-value < 0.05 was considered statistically significant for comparisons.

### Nucleotide Sequence Accession Numbers

The nucleotide sequences of the *qacH*-carrying integrons have been submitted to GenBank under accession numbers KY662007 for *aadA2-cmlA1-aadA1-qacH-*IS*440-sul3* and KY426918 for *dfrA32*-*ereA1*-*aadA2-cmlA1-aadA1-qacH-*IS*440-sul3*.

## Results

### Isolation of *P. mirabilis*

Colony characteristics of *P. mirabilis* on XLD agar were similar to those of *Salmonella*. In this study, *Salmonella*-like colonies were found in 88 samples (Supplementary Table S1). Among these samples, fifty-six samples were positive for isolates with swarming phenotype (Supplementary Table S1). These suspected *P. mirabilis* isolates were identified by the biochemical tests and PCR-based 16S rDNA sequencing. Fifty-two samples were positive for *P. mirabilis*, five samples were positive for *P. vulgaris*, and one sample was positive for *Salmonella* (Supplementary Table S1).

### Susceptibility of *P. mirabilis* Isolates to BC

**FIGURE 1:**
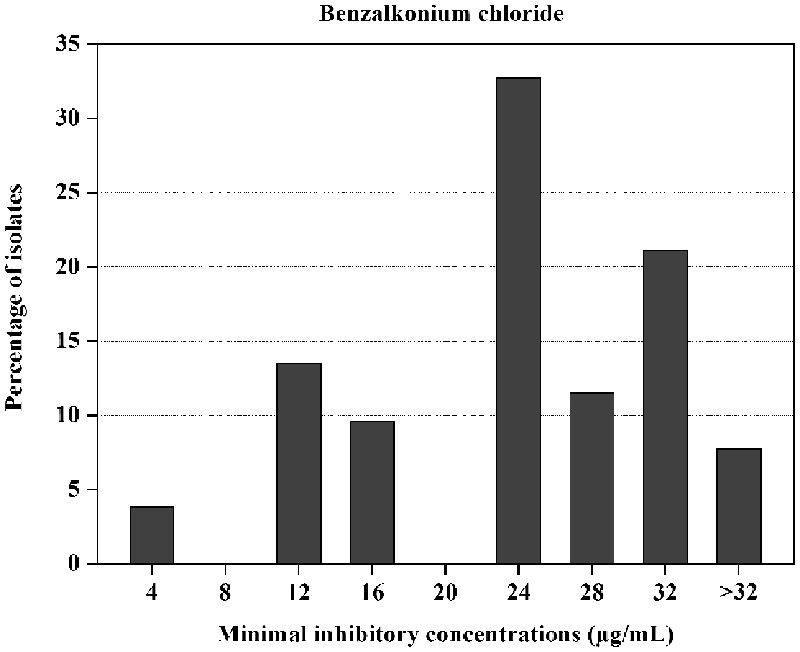
Distribution of the MICs of benzalkonium chloride for 52 *Proteus mirabilis* isolates.

A total of 52 (29.2%) *P. mirabilis* isolates were recovered from 178 cooked meat samples (Supplementary Table S1). For these isolates, the MICs of BC ranged from 4 to >32 μg/mL (**Figure 1** and Supplementary Table S2). The isolates with BC MICs of 24 μg/mL (*n* = 17, accounting for 32.7% of all isolates) were observed most frequently, followed by the isolates with MICs of 32 μg/mL (*n* = 11, 21.1%).

### Presence of QAC Resistance Genes

The presence of QAC resistance genes among *P. mirabilis* isolates are presented in Supplementary Table S2. The *mdfA* gene was the most widespread QAC resistance gene, being found in 100% of isolates, followed by *ydgE*/*ydgF* (90.4%, *n* = 47), *qacE* (53.8%, *n* = 28), *qacE*Δ1 (53.8%, *n* = 28), *emrE* (44.2%, *n* = 23), *sugE(c)* (40.4%, *n* = 21), *sugE(p)* (32.7%, *n* = 17), and *qacH* (3.8%, *n* = 2). The *qacF* and *qacG* genes were not detected in any of the isolates. The top two QAC resistance genotypes were *qacE-qacE*Δ1*-mdfA-ydgE/ydgF* (26.9%) and *mdfA-ydgE/ydgF* (13.5%) (**Table 2**).

**Table 2:** Different resistance gene combinations in *Proteus mirabilis* isolates.

Gene combination	Number of isolates with MIC (μg/mL) as follows							Total
	4	12	16	24	28	32	>32	
*qacE-qacE*Δ1*-mdfA-ydgE/ydgF*		4	1	9				14
*mdfA-ydgE/ydgF*	2	3	2					7
*sugE(p)-qacE-qacE*Δ1*-emrE-mdfA-ydgE/ydgF*				1	1	4		6
*sugE(c)-sugE(p)-qacE-qacE*Δ1*-emrE-mdfA-ydgE/ydgF*						2	2	4
*sugE(c)-qacE-qacE*Δ1*-emrE-mdfA-ydgE/ydgF*				3	1			4
*emrE-mdfA-ydgE/ydgF*			2	1	1			4
*sugE(c)-sugE(p)-mdfA*					1	3		4
*sugE(c)-emrE-mdfA-ydgE/ydgF*				2	1			3
*sugE(c)-sugE(p)-emrE-mdfA-ydgE/ydgF*						2		2
*sugE(c)-mdfA-ydgE/ydgF*				1	1			2
*sugE(c)-qacH-mdfA-ydgE/ydgF*							1	1
*sugE(c)-sugE(p)-qacH-mdfA*							1	1

Our results showed that *qacE* always occurred simultaneously with *qacE*Δ1, but the MICs of BC were not significantly different (*P* > 0.05) between *qacE*-*qacE*Δ1-positive and -negative isolates. The presence of *sugE(p)* was significantly associated with the higher MICs of BC (*P* < 0.05). Among the 17 *sugE(p)*-positive isolates, 82.4% (*n* = 14) had the MICs of ≥32 μg/mL. High MICs of BC (>32 μg/mL) were also observed in the two isolates that carried *qacH*.

### Genetic Structure of Class 1 Integrons

**FIGURE 2:**
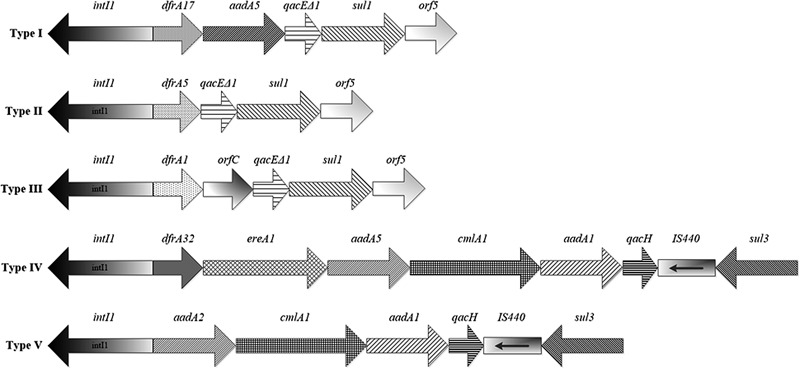
Schematic representation of the variable regions of class 1 integrons identified in *P. mirabilis* isolates. Integron type with different cassette arrays (Type I to V) are arranged as identified in **Table 3**.

The 52 *P. mirabilis* isolates were subjected to the PCR screening for the expected integrase gene, and the 565-bp corresponding amplicon was detected in 10 isolates, consistent with the presence of the class 1 integrase gene. Five groups of resistance gene cassettes, named as type I-V, were identified in these isolates (**Figure 2**), including: *dfrA17-aadA5, dfrA5, dfrA1-orfC, dfrA32*-*ereA1*-*aadA2*, and *aadA2* (**Table 3**).

**Table 3:** Characteristics of isolates carrying integrons.

Isolate	Class 1 integrons				
	Size (bp)	Gene cassette	Type^a^	*qacE*Δ1-*sul1*	Classic or non-classic^b^
PM1	1664	*dfrA17*-*aadA5*	I	_+_	Classic
PM4	2900	*dfrA32*-*ereA*-*aadA2*	IV	-	Non-classic
PM7	721	*dfrA5*	II	_+_	Classic
PM9	1664	*dfrA17*-*aadA5*	I	_+_	Classic
PM13	1664	*dfrA17*-*aadA5*	I	_+_	Classic
PM19	792	*aadA2*	V	-	Non-classic
PM21	1163	*dfrA1*-*orfC*	III	_+_	Classic
PM24	721	*dfrA5*	II	_+_	Classic
PM35	1664	*dfrA17*-*aadA5*	I	_+_	Classic
PM39	1664	*dfrA17*-*aadA5*	I	_+_	Classic

*^a^Type I-V represented five different integron structures in our study (**Figure 2**).*

*^b^In this study, class 1 integrons with *qacE*Δ1 and *sul1* genes in their 3′-CS were named as classic class 1 integrons. Accordingly class 1 integrons without the normal 3′-CS were named as non-classic class 1 integrons.*

### Analysis of Plasmids Carrying *qacH*-Associated Class 1 Integrons

**FIGURE 3:**
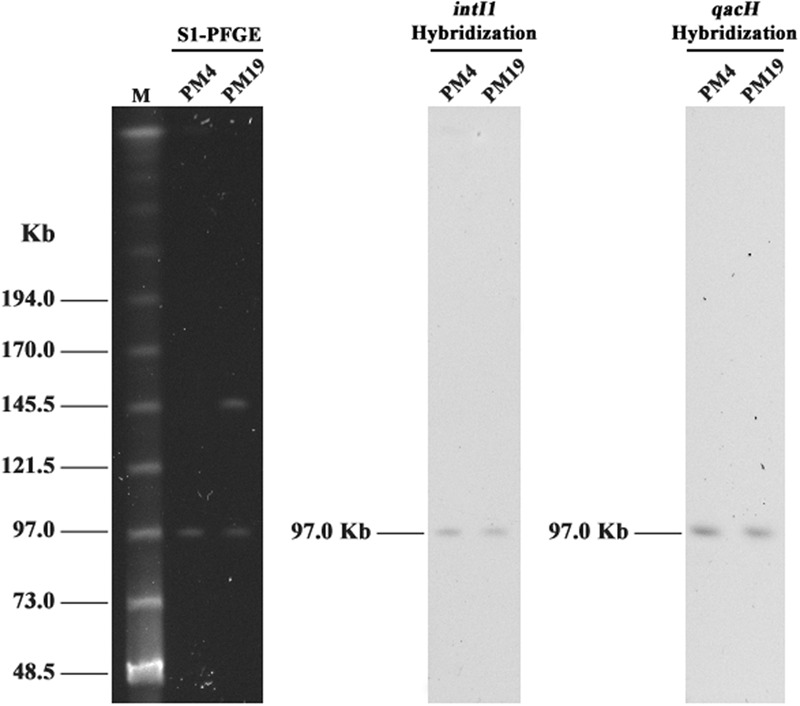
S1-PFGE and southern hybridization of *intI1* and *qacH* on two *qacH*-positive isolates. M, MidRange PFG Marker; PM4 and PM19 are *qacH*-positive *P. mirabilis* isolates.

According to the bands obtained by S1-PFGE of *qacH*-positive isolates, the number and size of their plasmids were determined (**Figure 3**). PM4 contained a plasmid of 100 kb and PM19 contained two large plasmids of 100 and 150 kb. Both specific probes for *intI1* and *qacH* genes hybridized with the plasmids of 100 kb in the two isolates (**Figure 3**). Two transconjugants named PM4T and PM19T were obtained after conjugation experiments. Both of them exhibited resistance to streptomycin and chloramphenicol. However, increased MICs of BC were not observed in transconjugants (data not shown). PCR experiments confirmed that the transconjugants harbored the same gene structure of integrons as their donors (PM4T with *dfrA32*-*ereA1*-*aadA2-cmlA1-aadA1-qacH-*IS*440-sul3* and PM19T with *aadA2-cmlA1-aadA1-qacH-*IS*440-sul3*). All the above indicated results confirmed that *qacH*-associated class 1 integrons in the two studied isolates were located on conjugative plasmids of 100 kb.

## Discussion

In the present study, fifty-two isolates of *P. mirabilis* were isolated from cooked meat samples. According to Wong et al. (2013), the rate of *P. mirabilis* in raw chicken carcass samples in Hong Kong was 86.2% (50/58), which was much higher than that (29.2%) in our study. Cooking procedures kill most microorganisms that colonize raw meats, however, survival of microorganisms due to improper processing or cross-contamination of food after cooking may occur. This could be an explanation for the lower incidence of *P. mirabilis* in cooked meat products compared to raw meat samples.

*Proteus mirabilis* isolates in our study showed the MICs of BC ranging from 4 to >32 μg/mL, with MICs of 24 μg/mL most frequently. As there is no standard resistant breakpoint of BC for *P. mirabilis*, it is difficult to classify the isolates as BC susceptible, intermediate or resistance in this study. Although data on the BC susceptibility in *P. mirabilis* were scarce, several studies have reported the MICs of BC in Enterobacteriaceae isolates from different sources (Sidhu et al., 2002; Aarestrup and Hasman, 2004; Zhang et al., 2016). In the study of Zhang et al. (2016), *E. coli* isolated from retail meat showed the MICs of BC in the range of 16–64 μg/mL. Aarestrup and Hasman (2004) have shown that the MICs of BC for *Salmonella* (*n* = 156) and *E. coli* (*n* = 202) from food animals ranged from 64 to 256 μg/mL and from 16 to 128 μg/mL, respectively. Compared with the studies mentioned above, *P. mirabilis* isolates in our study showed relatively lower MICs to BC. In another survey of Enterobacteriaceae from food, almost half of the isolates exhibited the MICs of BC with <10 μg/mL (Sidhu et al., 2002). Actually, the user concentrations of BC in food industry are usually 200–1000 μg/mL (Møretrø et al., 2017), which are much higher than the MICs of *P. mirabilis* in this study. In practical application, BC is commonly rinsed from surfaces (equipment, machines, floor etc.) with water after disinfection. This rinsing step, however, is not sufficient to remove all BC residues from surfaces and consequently, bacteria are likely to be exposed to diluted, sub-lethal BC concentrations (Buffet-Bataillon et al., 2012). Repeated exposure to sub-lethal BC concentrations may facilitate the development of resistance (Buffet-Bataillon et al., 2012). Therefore, it was not surprising that *P. mirabilis* isolates in the present study exhibited low-level of BC MICs.

In this study, the presence of QAC resistance genes was investigated. Our results showed that *mdfA* and *ydgE/ydgF* were the most prevalent among *P. mirabilis*, which was in agreement with the similar studies of *E. coli* (Zou et al., 2014; Zhang et al., 2016). Among the isolates tested in our study, *qacE* always occurred simultaneously with *qacE*Δ1. It was noted that the presence of *sugE(p)* was significantly associated with the higher MICs of BC (*P* < 0.05). Two *qacH*-positive isolates also exhibited relatively high MICs of BC (>32 μg/mL). In previous research, the higher MICs of BC were associated with plasmid-encoded genes (Zou et al., 2014). Because each isolate harbored more than one QAC resistance gene, it is difficult to assess what level of BC resistance was contributed by each QAC resistance gene.

A previous study reported that *aadA1* gene cassette was observed most commonly among the *P. mirabilis* isolates from retail meat products (Kim et al., 2005). In contrast, our results showed that *dfrA17-aadA5* was the most common cassette array, which is similar to other reports of foodborne *P. mirabilis* isolates in China (Shen et al., 2011). Interestingly, an uncommon integron gene cassette array *dfrA32*-*ereA1*-*aadA2* was found in one *P. mirabilis* isolate in this study. To the best of our knowledge, this is the first report of *dfrA32*-*ereA1*-*aadA2* in foodborne *P. mirabilis*. The integron gene cassette array *dfrA32*-*ereA1*-*aadA2* in our study showed 99.6% identity to that of *Salmonella enterica* (Krauland et al., 2010; GenBank accession number GU067642), 99.7% identity to that of *Laribacter hongkongensis* (Feng et al., 2011; GenBank accession number GU726907) and 99.7% identity to that of *Aeromonas hydrophila* (unpublished; GenBank accession number KJ543558). Recently, this cassette array was detected in clinical *P. mirabilis* isolates in Zhejiang Province of China (Wei et al., 2014), which had 99.6% identity to our sequence. The high similarity of *dfrA32*-*ereA1*-*aadA2* indicated that the class 1 integrons carrying this cassette array may be located on conjugative plasmids.

Classic class 1 integrons are composed of *qacE*Δ1 and *sul1* genes in their 3′-CS. Recently, class 1 integrons without the typical 3′-CS have been found in some species of Enterobacteriaceae, including *Escherichia coli, Salmonella, Shigella sonnei* and *Klebsiella pneumoniae* (Antunes et al., 2007; Chang et al., 2009, 2011; Sáenz et al., 2010). In these previous studies, the 3′-CS of integrons was associated with *qacH* and *sul3*. Xu et al. (2009) reported that two clinical isolates of *Pseudomonas aeruginosa* carried a class 1 integron lacking 3′-CS, however, the genetic structure of these non-classic integrons was unclear. In our study, non-classic class 1 integrons lacking *qacE*Δ1 and *sul1* genes were found in 2 of the 10 *intI1*-positive isolates (PM4 with *dfrA32*-*ereA1*-*aadA2* and PM19 with *aadA2*). PCR amplifications revealed that the gene cassettes in the two isolates were followed by an unusual 3′-CS linked to *qacH* and *sul3*. The gene cassette organization *dfrA32*-*ereA1*-*aadA2-cmlA1-aadA1-qacH-*IS*440-sul3* and *aadA2-cmlA1-aadA1-qacH-*IS*440-sul3* were identified in PM4 and PM19, respectively. Both of them contained *cmlA1* coding for chloramphenicol resistance, *aadA1* coding for streptomycin resistance, *qacH* coding for QAC resistance, insertion element IS*440*, and *sul3* coding for sulfonamide resistance. It seems that *sul3* had replaced *sul1* in addition to the replacement of *qacE*Δ1 by *qacH* in the 3′-CS region. Although the gene structure *cmlA1*-*aadA1*-*qacH*-IS*440*-*sul3* was commonly found in non-classic integrons (Antunes et al., 2007; Sáenz et al., 2010), the combination of *dfrA32*-*ereA1*-*aadA2-cmlA1-aadA1-qacH-*IS*440-sul3* was reported for the first time. This novel arrangement detected in *P. mirabilis* isolate from food has been included in GenBank with the accession number KY426918. A similar gene cassette array of *qacH-dfrA32*-*ereA1*-*aadA2-cmlA1-aadA1* was detected in *Salmonella enterica* serovar Stanley isolates from Taiwan (Krauland et al., 2010). Both of the two arrays carried the same gene cassettes, but the position of *qacH* was different. PM4 and PM19 had similar *qacH*-IS*440*-*sul3*-integron platforms with different gene cassette arrays, suggesting evolution of the genetic background by different recombinatorial events.

Our results demonstrated that non-classic class 1 integrons were located on conjugative plasmids of 100 kb in two tested isolates. Previous studies showed that the class 1 integrons lacking the normal 3′-CS and associated with *qacH* and *sul3* genes were usually located on large plasmids of different size (between 70 and 240 kb) and the conjugative plasmids of 100 kb were the most disseminated among the different isolates (Antunes et al., 2007; Sáenz et al., 2010). Pal et al. (2015) also found that plasmids with co-selection potential for resistance to disinfectants and antimicrobials tended to be large and conjugative. Conjugation experiments confirmed that the disinfectant and antimicrobial resistance genes of non-classic class 1 integrons in PM4 and PM19 were co-existed on the same conjugative plasmids and could be co-transferred to *E. coli*. When theses isolates are exposed to QACs, selection pressure from disinfectants could increase risks for the spread of QAC and antimicrobial resistance genes among the bacteria. Notably, two transconjugants containing *qacH* showed the same MICs of BC as the recipient in this study. Similar observations that the presence of QAC resistance genes didn’t increase the MICs of BC in transconjugants has also been reported in a previous study (Zhang et al., 2016). There was the possibility that the agar dilution method used for susceptibility testing was not sensitive enough to detect the differences of BC MICs between the recipient and transconjugants.

## Conclusion

Quaternary ammonium compounds resistance genes, including *mdfA, ydgE*/*ydgF, qacE, qacE*Δ1, *emrE, sugE(c)*, and *sugE(p)* were found in foodborne *P. mirabilis* isolates in this study. Our data demonstrated the presence of non-classic class 1 integrons with the gene structure *qacH*-IS*440*-*sul3* among the isolates. Moreover, *qacH*-associated non-classic class 1 integrons were located on conjugative plasmids and therefore could constitute an effective way for co-dissemination of antimicrobial and disinfectant resistance genes.

## Author Contributions

TY, HW, and LS designed and supervised the study. XJ, LL, YL, and KZ performed the experiments. XJ analyzed data. XJ and TY drafted the manuscript.

## Conflict of Interest Statement

The authors declare that the research was conducted in the absence of any commercial or financial relationships that could be construed as a potential conflict of interest.

## Footnotes

1 http://www.ncbi.nlm.nih.gov/BLAST/
